# Developmental and Post-Eruptive Defects in Molar Enamel of Free-Ranging Eastern Grey Kangaroos (*Macropus giganteus*) Exposed to High Environmental Levels of Fluoride

**DOI:** 10.1371/journal.pone.0147427

**Published:** 2016-02-19

**Authors:** Uwe Kierdorf, Clare Death, Jasmin Hufschmid, Carsten Witzel, Horst Kierdorf

**Affiliations:** 1 Department of Biology, University of Hildesheim, Hildesheim, Germany; 2 Faculty of Veterinary and Agricultural Sciences, The University of Melbourne, Werribee, Victoria, Australia; Max Planck Institute for Evolutionary Anthropology, GERMANY

## Abstract

Dental fluorosis has recently been diagnosed in wild marsupials inhabiting a high-fluoride area in Victoria, Australia. Information on the histopathology of fluorotic marsupial enamel has thus far not been available. This study analyzed the developmental and post-eruptive defects in fluorotic molar enamel of eastern grey kangaroos (*Macropus giganteus*) from the same high-fluoride area using light microscopy and backscattered electron imaging in the scanning electron microscope. The fluorotic enamel exhibited a brownish to blackish discolouration due to post-eruptive infiltration of stains from the oral cavity and was less resistant to wear than normally mineralized enamel of kangaroos from low-fluoride areas. Developmental defects of enamel included enamel hypoplasia and a pronounced hypomineralization of the outer (sub-surface) enamel underneath a thin rim of well-mineralized surface enamel. While the hypoplastic defects denote a disturbance of ameloblast function during the secretory stage of amelogenesis, the hypomineralization is attributed to an impairment of enamel maturation. In addition to hypoplastic defects, the fluorotic molars also exhibited numerous post-eruptive enamel defects due to the flaking-off of portions of the outer, hypomineralized enamel layer during mastication. The macroscopic and histopathological lesions in fluorotic enamel of *M*. *giganteus* match those previously described for placental mammals. It is therefore concluded that there exist no principal differences in the pathogenic mechanisms of dental fluorosis between marsupial and placental mammals. The regular occurrence of hypomineralized, opaque outer enamel in the teeth of *M*. *giganteus* and other macropodids must be considered in the differential diagnosis of dental fluorosis in these species.

## Introduction

Dental enamel is the hardest tissue of the mammalian body, with a mineral (impure hydroxyapatite) proportion of 96–98% by weight in the mature tissue [1–4]. Enamel is produced by ameloblasts, which form by differentiation of the cells of the inner enamel epithelium and are tightly connected to each other via junctional complexes [1, 5, 6].

Enamel formation (amelogenesis) can be broadly divided into a secretory stage and a maturation stage. During the secretory stage, the ameloblasts synthesize and secrete a collagen-free proteinaceous matrix. Immediately after its secretion, long thin mineral ribbons of amorphous calcium phosphate (later maturing into hydroxyapatite crystals) form within this matrix, a process that is controlled by specific matrix proteins [6–8]. The enamel of mammals is characterized by the occurrence of bundles of closely packed hydroxyapatite crystals referred to as prims or rods. Each prism is formed in relation to a secretory surface at the distal pole of the Tomes’ process, an extension of the cell body that characterizes secretory-stage ameloblasts [1, 5, 6]. The activity of secretory ameloblasts fluctuates in a cyclic fashion, and the resulting variation of enamel growth rate is reflected by the occurrence of regular incremental markings that are preserved in the mature tissue [1, 8–16].

When the full thickness of enamel is reached at a given location of the tooth crown, the ameloblasts go through a brief transitional phase and enter the maturation stage. The principal functions of maturation-stage ameloblasts are the production and release of enzymes (kallikrein-4 and matrix metalloproteinase-20) that degrade enamel matrix proteins, the absorption of the degradation products and of water, and the movement of calcium, phosphate and bicarbonate ions into the matrix [6, 8]. In consequence, the crystals formed in the secretory stage further increase in width and thickness until the final high mineral content of mature enamel is reached [6, 8, 17, 18]. Bicarbonate ions are required to neutralize the H^+^-ions generated by hydroxyapatite formation [19]. During the maturation stage, the ameloblasts cyclically change between two (ruffle-ended and smooth-ended) morphologies, a phenomenon referred to as ameloblast modulation [5, 6, 17].

After completion of amelogenesis, the ameloblasts become reduced in size and form a protective layer covering the enamel surface. Pre-eruptive disintegration of this so-called reduced enamel epithelium is followed by the deposition of cementum onto the enamel, a process that regularly occurs in various mammal species [3]. However, deposition of coronal cementum can also occur as a pathological phenomenon following premature breakdown of the enamel organ in species that do not normally possess a cementum covering of their tooth crowns [20].

Since it constitutes a cell-free, highly mineralized extracellular matrix, enamel is incapable of undergoing remodelling or cell-mediated repair processes. Aberrations in the structure and volume of enamel caused by an impairment of secretory ameloblast function therefore remain as a permanent record in the affected tooth [1, 21–23]. Microstructural defects include accentuated incremental markings and the occurrence of zones of aprismatic enamel. A reduction in the volume of enamel produced manifests as hypoplasia [22–26]. Impairment of the mineralization process results in enamel hypomineralization, the extension and severity of which depend on the onset, duration and intensity of the impact [21, 27, 28]. There is experimental evidence that minor mineralization defects in developing enamel can be “healed” during later stages of maturation to the extent that they are no longer visible in microradiographs [28, 29]. In contrast, more severe mineralization defects remain as a permanent record in the enamel.

Prolonged intake of excessive amounts of fluoride during tooth development causes pathological changes in enamel that are referred to as dental fluorosis or enamel fluorosis [28, 30–35]. In humans, dental fluorosis is mostly observed as a consequence of consumption of drinking-water high in fluoride [36, 37]. Another cause of widespread dental (and skeletal) fluorosis in humans is indoor coal burning [36, 38]. While the lesions of dental fluorosis can only develop during the period of tooth formation and thereafter remain as a permanent record in the dentition, skeletal fluorosis can be induced throughout life.

Dental fluorosis has been reported also from domestic animals exposed to excess dietary fluoride [36, 39–41] and in wild mammals living in areas with increased environmental levels of fluoride from natural [42–44] or anthropogenic sources [42, 45–54]. The developmental and post-eruptive defects of fluorotic enamel have been studied in detail in humans and rodents [27, 28, 31, 32, 55–62]. Further studies characterized fluorotic enamel in domestic sheep [63–65], cattle [66] and pigs [67, 68], as well as in free-ranging wild boar [20, 53] and deer [48, 50, 69–71].

Recently, increased bone fluoride concentrations and occurrence of dental and skeletal lesions diagnosed as dental and skeletal fluorosis, respectively, were reported from free-ranging marsupials inhabiting the surroundings of an aluminium smelter in Portland, Victoria, Australia [72–75]. The macroscopic lesions recorded in the fluorotic marsupial teeth [74] closely resemble those previously reported for teeth of placental herbivores from fluoride-polluted areas [42, 44, 48, 50, 51]. In the marsupials from the high-fluoride area, teeth whose crowns formed entirely or largely before weaning displayed either no or only minor fluorotic lesions, while later-forming teeth regularly showed more severe degrees of dental fluorosis [74].

Thus far, information on the histopathological features of fluorotic marsupial enamel has been lacking. The present study, therefore, analyzed the developmental and post-eruptive defects in fluorotic enamel of a marsupial species, the eastern grey kangaroo (*Macropus giganteus*) from the high-fluoride area in Portland, using light and scanning electron microscopy. The results are compared to those previously reported for placental mammals to check whether, in addition to the macroscopic appearance, the microstructural features of fluorotic marsupial enamel also correspond to those observed in placental mammals.

## Materials and Methods

### Study areas

The research reported in this manuscript was conducted according to all relevant Australian laws and guidelines and carried out under Department of Sustainability and Environment (Victoria, Australia) Wildlife Research and Management permits. Study areas included private land and were accessed with permission of the owner, Alcoa Portland Aluminium Pty Ltd. Future researchers must contact Alcoa Portland Aluminium Pty Ltd. for access to these sites.

The high-fluoride area exists within a buffer zone of approximately 600 hectares around the Portland Aluminium smelter situated on the southern headland of Portland Bay, Victoria, Australia at 38°23’ S, 141°37’ E. The area contains dry and coastal heathland, small wetlands and farmland [76] and is inhabited by a community of marsupial species, including the eastern grey kangaroo (*Macropus giganteus*) [74].

**Fig 1:**
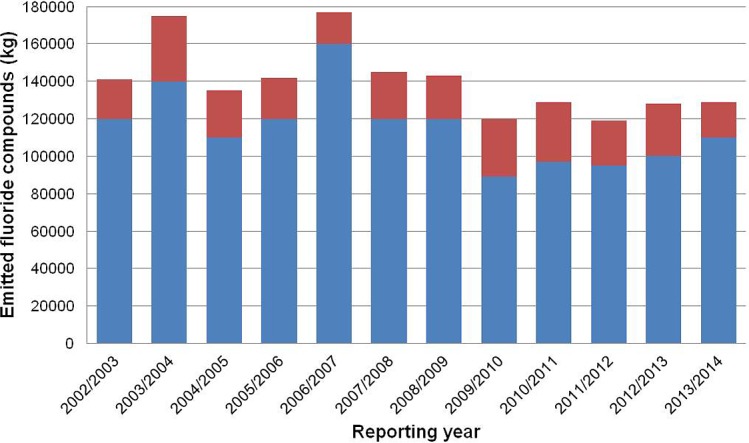
Emissions of fluoride compounds (kg/year) from the Portland Aluminium smelter (blue) and the Incitec fertilizer plant (red) for the period 2002/2003 through 2013/2014 according to data from the NPI [77]. Reporting years range from July 1 to June 30 of the following year.

The Portland Aluminium smelter (run by Alcoa Portland Aluminium Pty Ltd) started operations in 1986. During the reporting years 2002/2003 to 2013/2014 (periods from July 1 to June 30 of the following year), total annual emissions of fluoride compounds from the Portland Aluminium smelter ranged between 89,000 and 160,000 kg (Fig 1) [77]. A further source of fluoride emissions affecting the area is a fertilizer works approximately 3km from the smelter site (Incitec Pivot Ltd. Portland, situated at 38°21’ S, 141°37’ E). During the reporting years 2002/2003 to 2013/2014, annual emissions of fluoride compounds from this point source varied between 17,000 and 35,000 kg (Fig 1) [77]. For comparison with animals from the high-fluoride area, we obtained individuals from low-fluoride areas in Victoria not impacted by emissions from the two point sources [74]. Fluoride is highly immobile in soil and plant uptake from the soil is therefore generally low, except for some species referred to as accumulators [36, 41]. Fluoride uptake by plants therefore occurs largely from the atmosphere, either by diffusion of gaseous fluoride into leaves or following dry or wet deposition of particulate fluorides on plant surfaces [36, 41]. Vegetation fluoride levels measured in the high-fluoride area of this study varied between 5 and 600 μg/g dry matter across seasons in the period 2008–2013 [unpublished data], while background fluoride concentrations in coastal native vegetation in Victoria, Australia has previously been shown to be generally below 10 μg/g dry matter [78].

### Study species

The eastern grey kangaroo is a large terrestrial herbivore that is distributed throughout eastern and south-eastern Australia and parts of Tasmania [79]. The species is generally classified as a grazer [80]. Body mass of eastern grey kangaroos mostly ranges between 20 and 37 kg in females and between 25 to 50 kg in males, although individual animals can be considerably heavier [80]. The heaviest male eastern grey kangaroo studied by one of us (JH, unpublished data) weighed 92 kg.

Fluoride intake in mammals occurs largely via food and water, while inhalation is considered to be of minor importance [41]. In herbivorous species, fluoride compounds deposited on plant surfaces have to be considered as a dietary source of fluoride, in addition to the fluoride incorporated in the plants [41]. In grazers like *M*. *giganteus*, the accidental uptake of soil particles during feeding constitutes a further potential exposure route. Geophagy, the intentional consumption of soil, has not been observed in intensively studied populations of *M*. *giganteus* from Victoria [81].

**Fig 2:**
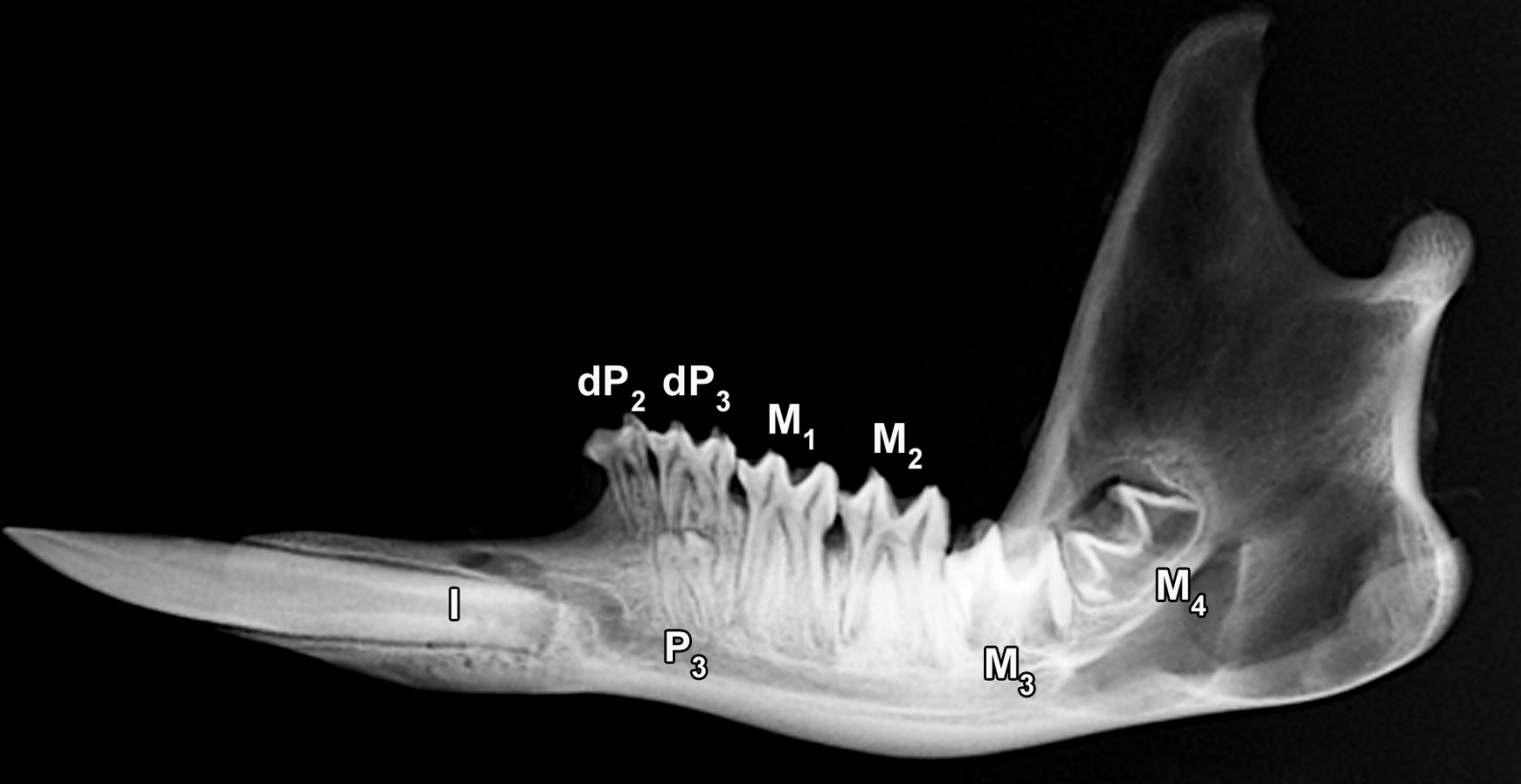
Lateral radiograph of the right mandible of an eastern grey kangaroo, *Macropus giganteus* (individual No.1153 (female), estimated age 2.3 yr) from the high-fluoride area. The radiograph shows the single large procumbent lower incisor (I) and the (erupted and unerupted) cheek teeth (premolars dP_2_, dP_3_ and P_3_, and molars M_1_ to M_4_). Of the cheek teeth, dP_2_, dP_3_, M_1_ and M_2_ are in function.

The dental formula of *M*. *giganteus* is given as I 3/1, C 0/0, P 2/2, M 4/4 [79, 82]. A fifth molar can occasionally be present as a supernumerary tooth [82]. The designation of the premolars in the literature is inconsistent [82, 83]. According to Luckett [83], whose view is adopted here, there are two deciduous premolars (dP2 and dP3) in each jaw quadrant, and the single replacing (permanent) premolar is the P3. The four molars (M1 to M4) in each quadrant erupt sequentially at the posterior ends of the dental arcades, and gradually move anteriorly with age [79, 82]. This so-called molar progression occurs along with the sequential shedding of the tooth located most anteriorly within the cheek-tooth row. The radiograph of a right mandible of *M*. *giganteus* (Fig 2) shows the single large mandibular incisor (the I_2_ according to [82]) and the (erupted and unerupted) premolars and molars in a female with an estimated age of 2.3 years.

Kangaroo teeth possess tubular enamel [84–87], as is with few exceptions (Vombatidae and a few other taxa) typical for marsupials [88]. The enamel tubules are continuous with dentinal tubules, and in *Macropus* occur at a high density in the inner and central enamel [85–87, 89, 90]. Towards the enamel surface, the number of tubules sharply decreases, and in *Macropus* no tubules reach the unworn enamel surface [85, 87, 90]. Accordingly, the outer enamel region is referred to as atubular. The enamel of kangaroo teeth is regularly covered by cellular coronal cementum [89]

In the present study, the mandibular dentition of nineteen eastern grey kangaroos (14 females, 5 males) from the high-fluoride area and of eight individuals (1 female, 7 males) from the low-fluoride areas was initially inspected macroscopically (Table 1). All animals from the high-fluoride area exhibited macroscopic lesions of dental fluorosis in some of their teeth [74], whereas the teeth of the kangaroos from the low-fluoride areas were free from such lesions. The carcasses from which the analyzed teeth originated were collected in the period 2009 to 2012 and represented road-killed animals or individuals culled for population control. The carcasses were kept frozen until necropsy, when body condition was scored, standard external morphometric data were recorded, and a gross assessment of internal organs was performed [74]. Skulls and major postcranial skeletal elements (for details see [75]) were subsequently defleshed by manual dissection, macerated and cleaned. Age estimation in the animals was performed using a molar index [91]. This method is based on molar progression and assesses the position of the maxillary molars relative to a reference location in cleaned crania. Following necropsy, one cleaned and dried mandible (or mandibular fragment with cheek teeth) per animal was sent to the laboratory in Hildesheim for further study. Upon arrival, the mandibles were first immersed in 70% ethanol for a week, cleaned from remaining adhering soft tissue, thoroughly rinsed with water and air-dried. The teeth themselves were not cleaned to prevent damage to the enamel. The studied material (mandibles, teeth and tooth sections) has been deposited in the specimen collection of the Department of Biology, University of Hildesheim.

**Table 1:** Overview of the eastern grey kangaroos, *Macropus giganteus*, (*n* = 27) included in this study. The animals originated either from a high-fluoride area around the Portland aluminium smelter (Portland smelter site) or from different low-fluoride areas.

Identification number of individual^1^	Location	Year^2^	Sex	Age at death (yrs)^3^	Bone fluoride concentration (μg/g dry wt.)^4^
1000	Portland smelter site	2009	female	2.8	7385
1001	Portland smelter site	2011	female	5.2	3003
1007	Portland smelter site	2010	female	6.8	3845
1019	Portland smelter site	2010	female	4.5	2994
1020	Portland smelter site	2010	female	4.5	1246
1023	Portland smelter site	2011	male	1.8	662
1101	Portland smelter site	2009	female	12.5	6829
1109	Portland smelter site	2009	female	11.6	4408
1123	Portland smelter site	2009	female	1.6	1664
1153	Portland smelter site	2009	female	2.3	5527
1154	Portland smelter site	2009	female	5.9	6024
1163	Portland smelter site	2009	female	3.4	1175
1168	Portland smelter site	2009	male	13.3	2660
1198	Portland smelter site	2009	female	3.7	3668
1228	Portland smelter site	2011	female	13.3	7773
1259	Portland smelter site	2011	female	4.2	5699
1414	Portland smelter site	2012	male	4.8	2491
1441	Portland smelter site	2012	female	5.5	3546
1455	Portland smelter site	2012	male	11.6	2552
**2**	Grampians National Park	2009	female	5.2	62
**1248**	Lower Glenelg National Park	2011	male	4.5	75
**3003**	Tower Hill Wildlife Reserve	2011	male	2.3	99
**4238**	Woodlands Historic Park	2009	male	3.9	50
**4250**	Woodlands Historic Park	2009	male	3.0	79
**4253**	Woodlands Historic Park	2009	male	4.8	195
**4257**	Woodlands Historic Park	2009	female	3.4	50
**5050**	Cape Bridgewater	2011	male	2.0	123

^1^Bold numbers identify animals from the low-fluoride areas

^2^Year of collection of the carcasses

^3^Estimated using the molar index by Kirkpatrick [91]

^4^Except for individual No.2, for which the fluoride concentration in the mandible is reported, all fluoride values were determined in the calcaneus.

### Bone fluoride analysis

As a measure of fluoride exposure, bone fluoride concentration was determined in ashed samples (600°C for 12h, following drying at 105°C overnight) via the ion-selective electrode method [73]. Except for a single individual, the calcaneal bone was used for analysis. In one case (individual No. 2) this bone was not available, and fluoride concentration was measured in the mandible. Two replicate samples per bone were analyzed, and the mean of the two values is given and was used for statistical analysis. Bone fluoride concentrations are reported as micrograms per gram of dry bone. The analytical method was validated by analysis of duplicate samples at an independent laboratory (as detailed in [74]). A previous study showed that sex had no effect on bone fluoride concentrations in *M*. *giganteus* [73].

### Statistics

As some of the data (age in the animals from the high-fluoride area and bone fluoride concentration in the animals from the low-fluoride areas) did not meet the assumption of normal distribution, the rank-based non-parametric Mann-Whitney U-test was used to compare bone fluoride levels and age in the kangaroos from the two groups. Bone fluoride concentrations were compared to test the hypothesis that the animals from the high-fluoride area had a higher fluoride uptake during life than those from the low-fluoride areas. Age in the two groups was compared as a non-linear (more intense in young animals) increase in bone fluoride concentration with age had previously been demonstrated in *M*. *giganteus* from both high-and low-fluoride areas [73]. In the statistical tests a p-value < 0.05 was considered to indicate significance.

### Examination of teeth

The cheek tooth rows of the dry mandibles or mandibular fragments were macroscopically inspected and photographed with a digital camera (Canon EOS 300D, Canon Inc., Tokyo, Japan). Higher magnification images of individual teeth were captured with a digital reflected-light microscope (Keyence VHX-500F, Keyence Corp., Osaka, Japan) equipped with a high performance zoom lens (Keyence VH-Z20R; magnification range 20× to 200×).

To obtain samples for microscopic inspection, 16 molars from 12 individuals (9 from the high-fluoride area and 3 from low-fluoride areas) and the associated mandibular bone were sectioned out of the mandibles and embedded in epoxy resin (Biodur E12, hardener E1, Biodur products, Heidelberg Germany). The embedded specimens were subsequently bisected in an axiobuccolingual direction through either the anterior or the posterior tooth half. For imaging in the scanning electron microscope (SEM), the cut surface of one of the tooth halves was smoothed and polished using a series of silicon carbide papers (grits 320 to 2400). This was followed by sequential polishing on a motorized polisher (Labopol-5, Struers, Copenhagen) with diamond suspensions (Dia Pro, Struers) of nine and three μm particle diameter, respectively, and a final polishing step using a colloidal silica suspension (OP-S; Struers). The polished surfaces were examined uncoated in an SEM (Quanta 600 FEG, FEI Co., Hillsboro, OR, USA) fitted with a backscattered electron (BSE) detector, using an accelerating voltage of 20kV.

BSE imaging in the SEM is based on the detection of electrons from the primary electron beam accelerated into the specimen and backscattered in a surface layer after elastic collision with the atomic nuclei of the sample material. Electron backscattering increases with increasing atomic number of the elements in the sample [92]. In apatite-containing mineralized tissues, the concentration of calcium in the analyzed specimen is the major factor determining the amount of backscattered electrons. Variation in the intensity of the BSE signal is reflected by grey-level variation in the BSE images of the polished tooth sections, with brighter grey-levels corresponding to increased signal intensities and thus higher degrees of mineralization [93, 94]. Individual grey-scale images were converted to (pseudo-)colour images using the 16-colours lookup table of the software package ImageJ 1.46r (NIH, Bethesda, USA). For that, the 256 grey-level values from black (0) to peak white (255) were assigned to 16 bands of equal width, each represented by a different colour. For comparison, the distribution of grey-level values in selected areas was analyzed and displayed as histograms along with descriptive statistics.

For light microscopy, tooth halves prepared as described above were mounted with their polished sides on glass slides, using the epoxy resin as glue. The mounted specimens were sectioned to a thickness of about 300 μm, using an electric circular saw (Woko 50, Conrad Apparatebau GmbH, Clausthal-Zellerfeld, Germany) with a water-cooled diamond wafer blade. The sections were then ground and polished dry using silicon carbide papers (grits 320 to 4000), followed by a final polishing step using polishing wax (Menzerna, Ötigheim, Germany) and a leather cloth. The (unstained) ground sections, which had a thickness of about 50 μm, were cover-slipped and viewed and photographed in a Zeiss Axioskop2 Plus microscope (Carl Zeiss Microscopy GmbH, Oberkochen, Germany) equipped with a digital camera (Zeiss Axiocam 503 color). Imaging was performed using normal transmitted light (partly with phase contrast) or linearly polarized light with a full-wave (λ/1) retardation plate inserted between specimen and analyzer.

## Results

### Bone fluoride concentrations and age

Fluoride concentrations in the bones of the eastern grey kangaroos from the low-fluoride areas ranged between 50 and 195 μg/g dry wt, whereas the values for the animals from the high-fluoride area ranged between 662 and 7773 μg/g dry wt. Age ranged between 2.0 and 5.2 years in the animals from the low-fluoride areas and between 1.6 and 13.3 years in those from the high-fluoride area (Table 2) The difference in bone fluoride concentration between the two groups was highly significant (p < 0.0001), whereas no significant difference existed for age (p = 0.12) (Table 2).

**Table 2:** Bone fluoride concentration and age in eastern grey kangaroos (*Macropus giganteus*) from the low and high-fluoride areas.

Area		Bone fluoride (μg/g dry wt)^1^			Age (yrs)^2^	
	Mean	Median	Min., Max.	Mean	Median	Min., Max.
Low-fluoride (n = 8)	92	77	50, 195	3.6	3.7	2.0, 5.2
High-fluoride (n = 19)	3882	3546	662, 7773	6.3	4.8	1.6, 13.3

^1^Significant difference between the two groups (Mann-Witney U-test, p < 0.0001).

^2^ No significant difference between the two groups (Mann-Whitney U-test, p = 0.12).

### Macroscopic appearance of molars

**Fig 3:**
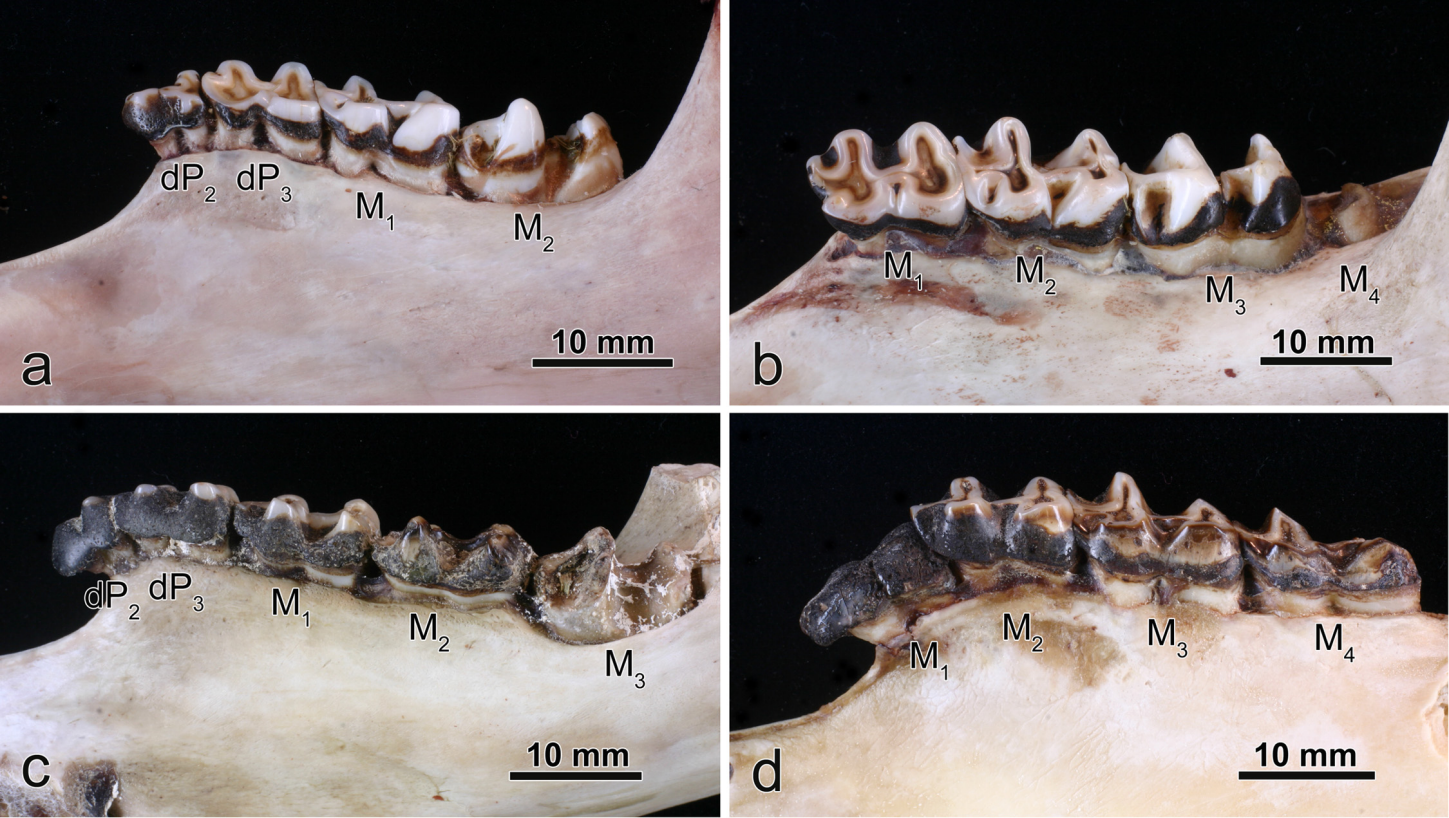
Macroscopic aspects of mandibular cheek-tooth rows of *Macropus giganteus* from low-fluoride areas (a,b) and the high-fluoride area (c,d). (a) Individual No.4238 (male), buccal view of left tooth row; (b) individual No.2 (female), buccal view of left tooth row; (c) individual No.1000 (female), lingual view of right tooth row; (d) individual No.1109 (female), buccal view of left tooth row. Note the normal whitish appearance of enamel and normal tooth wear in (a) and (b), and the brownish staining of enamel and abnormal wear of M_2_ and M_3_ in (c) and M_2-4_ in (d). Also note the dark brown to black dental calculus partially or entirely (M_1_ in (d)) covering the tooth crowns.

**Fig 4:**
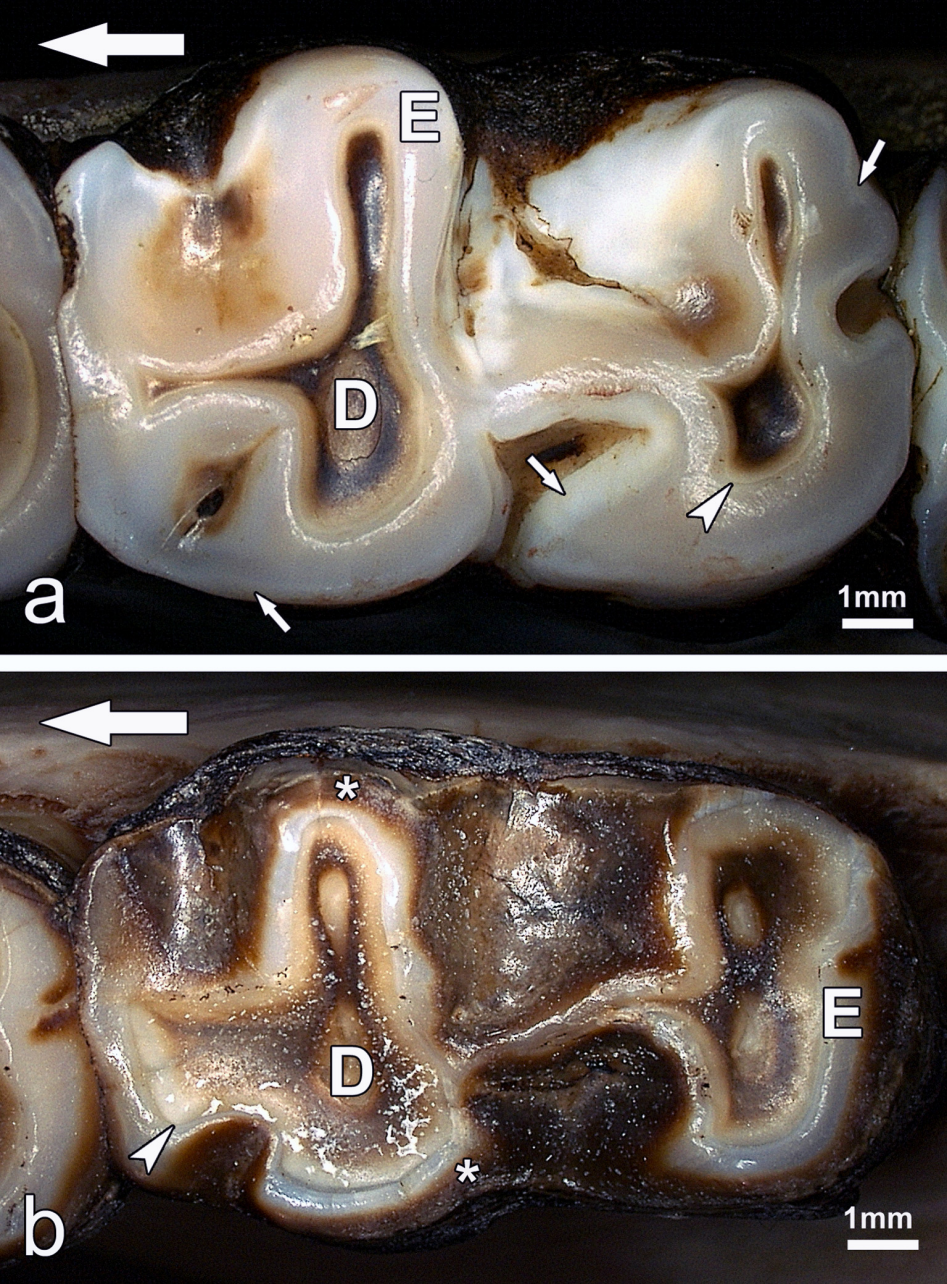
Mandibular molars of *Macropus giganteus* in occlusal view. (a) Left M_2_ of individual No.2 (female) from a low-fluoride area (control tooth); (b) left M_4_ of individual No.1109 (female) from the high-fluoride area (fluorotic tooth). In both teeth, enamel (E) and dentine (D) are exposed on the occlusal surface; the enamel-dentine junction (EDJ) is marked by arrowheads. Note translucent inner and more opaque outer enamel (small arrows) in (a) and brownish staining of outer enamel (asterisks) in (b). Large arrows indicate anterior direction.

**Fig 5:**
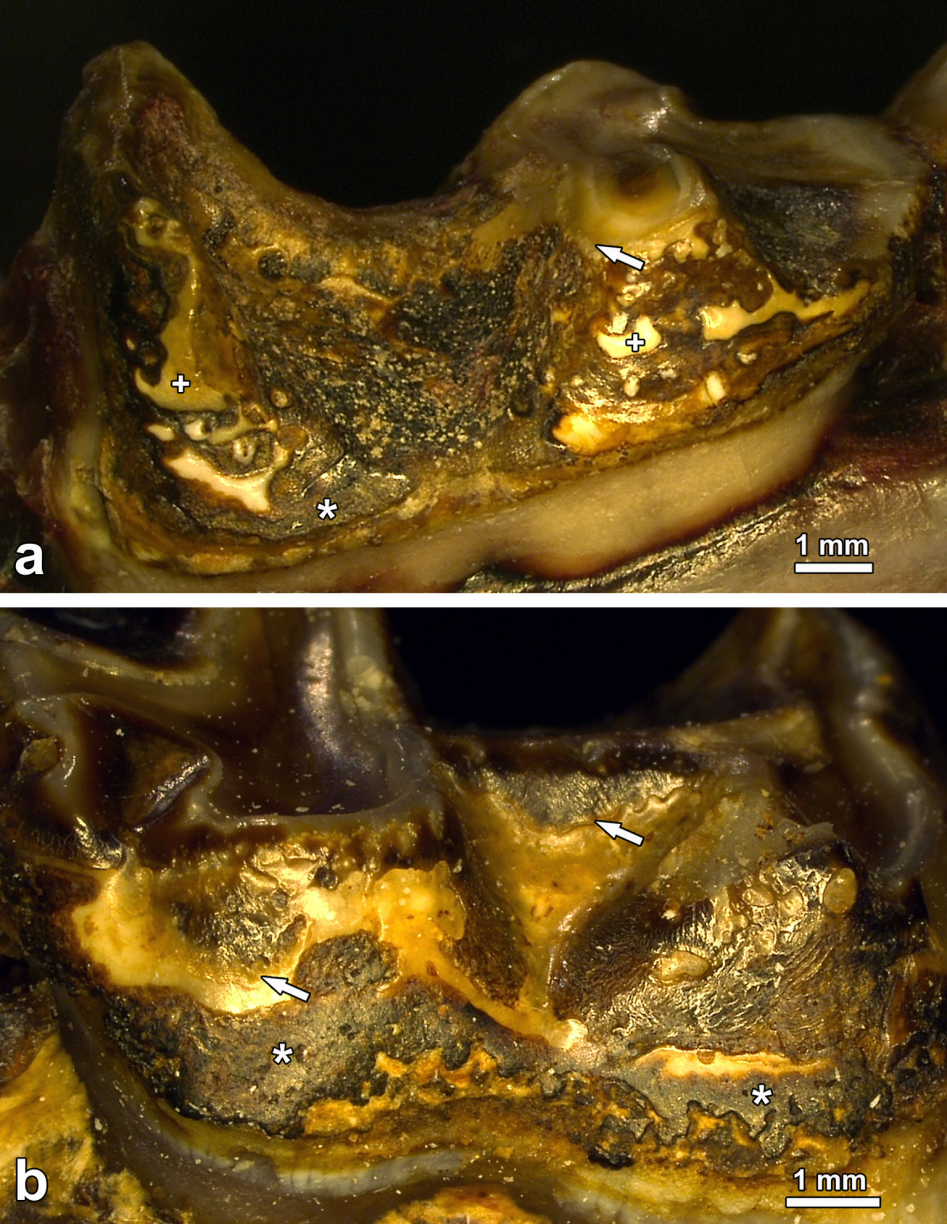
Fluorotic mandibular molars of *Macropus giganteus* from the high-fluoride area showing enamel surface defects and increased occlusal wear. (a) Left M_4_ of individual No. 1007 (female), lingual view, note abnormal reduction of enamel ridges and local loss of outer enamel (arrow) on the occlusal surface of the anterior tooth half and extensive surface defects on the crown flank with few areas of intact enamel remaining (crosses), asterisk: dental calculus; (b) left M_3_ of individual No. 1441 (female), buccal view, note reduction of enamel ridges on the occlusal surface, and large defect areas (arrows indicate cervical borders of defects) on the crown flank where portions of outer enamel have apparently flaked off post-eruptively, asterisks: dental calculus.

Molar enamel of the eastern grey kangaroos from the low-fluoride areas showed an overall whitish and glossy appearance (Fig 3A and 3B). On the occlusal surfaces, the enamel stood proud of the dentine, i.e., the enamel formed distinct ridges around dentinal troughs (Figs 3A, 3B and 4A). In contrast, molar enamel of the animals from the high-fluoride area exhibited varying degrees of brownish discolouration in the form of a light-brown to almost black staining as well as surface defects, indicative of either an incomplete formation of the enamel layer (enamel hypoplasia) or a post-eruptive enamel loss due to chipping or flaking away of portions of outer enamel (Figs 3C, 3D, 5A and 5B). Enamel discolouration was only seen in erupted portions of the tooth crown, indicating that it was due to infiltration of stains from the oral cavity. Molars showing enamel discolouration and surface defects also exhibited increased wear of the enamel ridges on their occlusal surface as well as an increased overall wear compared to teeth from the control individuals of similar age (Figs 3C, 3D, 4B, 5A and 5B). In severe cases, this resulted in a marked deviation from normal (age-related) crown shape of the molars (e.g., M_4_ in Fig 3D). Within tooth rows affected by fluorosis, the M_3_ and M_4_ typically showed the most pronounced lesions, while the M_1_ frequently appeared normal (Fig 3C) and the M_2_ was sometimes only moderately (Fig 3D), sometimes more severely affected (Fig 3C).

The enamel of the eastern grey kangaroos from the low-fluoride areas was divided into two zones that were readily distinguishable in the enamel exposed at the occlusal surface. An inner zone bordering on the enamel-dentine junction (EDJ) showed the whitish-translucent appearance typical for mature mammalian enamel, whereas the outer zone was composed of more opaque enamel (Fig 4A). The width of the opaque enamel zone varied between different areas of the tooth crown. The enamel of the eastern grey kangaroos from the high-fluoride area could likewise be divided into two zones. The enamel discolouration characteristic of the teeth was most pronounced in, and often confined to, the outer layer, whereas the inner zone bordering on the EDJ was of a distinctly more normal appearance (Fig 4B). Compared to the molar enamel of individuals from the low-fluoride areas, the opacity of the outer enamel in animals from the high-fluoride area was typically much more pronounced, sometimes resulting in a chalky appearance.

The crowns of the teeth from both groups were often covered with (dark brown to blackish) dental calculus to different degrees (Figs 3A, 3C, 3D, 5A and 5B). Within cheek tooth rows, the calculus deposits were most extensive in the more anteriorly located teeth (Fig 3C and 3D), and sometimes the whole tooth crown was covered with dental calculus (e.g. M_1_ in Fig 3D). This suggests that calculus build-up on the crowns increases with time of exposure to the oral environment and is especially pronounced in teeth that are no longer in occlusion.

### Microscopic characterization of normal and fluorotic enamel

**Fig 6:**
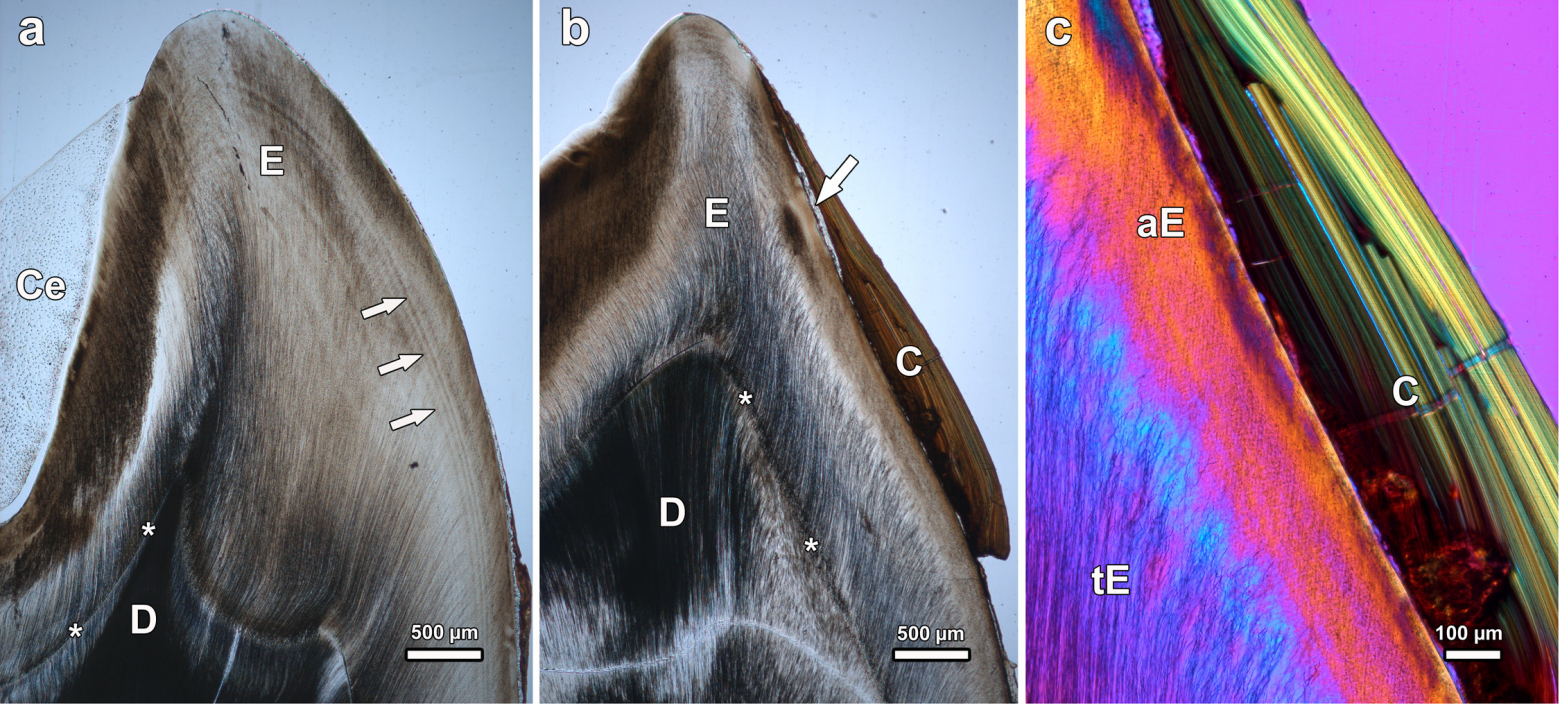
Light microscopic images of ground sections of mandibular molars of *Macropus giganteus* from low-fluoride areas, viewed in normal transmitted light (a, b) and in linearly polarized light with λ/1-plate (c). (a) Left M_3_ of individual No.4253 (male), axiobuccolingual section through the posterior tooth half. Note incremental lines (arrows) in the enamel (E) and filling of depressions in the crown surface with cellular cementum (Ce); D: dentine; asterisks: enamel-dentine junction; buccal to right of image. (b) Left M_3_ of individual No.2 (female), axiobuccolingual section through the anterior tooth half. Note thin patch of cementum (arrow) locally covering the enamel (E) on the buccal crown flank and more extended covering of the crown with dental calculus (C); D: dentine; asterisks: enamel-dentine junction; buccal to right of image. (c) Higher magnification of buccal enamel of the left M_3_ of individual No.2. Note inner tubular enamel (tE) and outer atubular enamel (aE), and distinct layering of the dental calculus (C).

**Fig 7:**
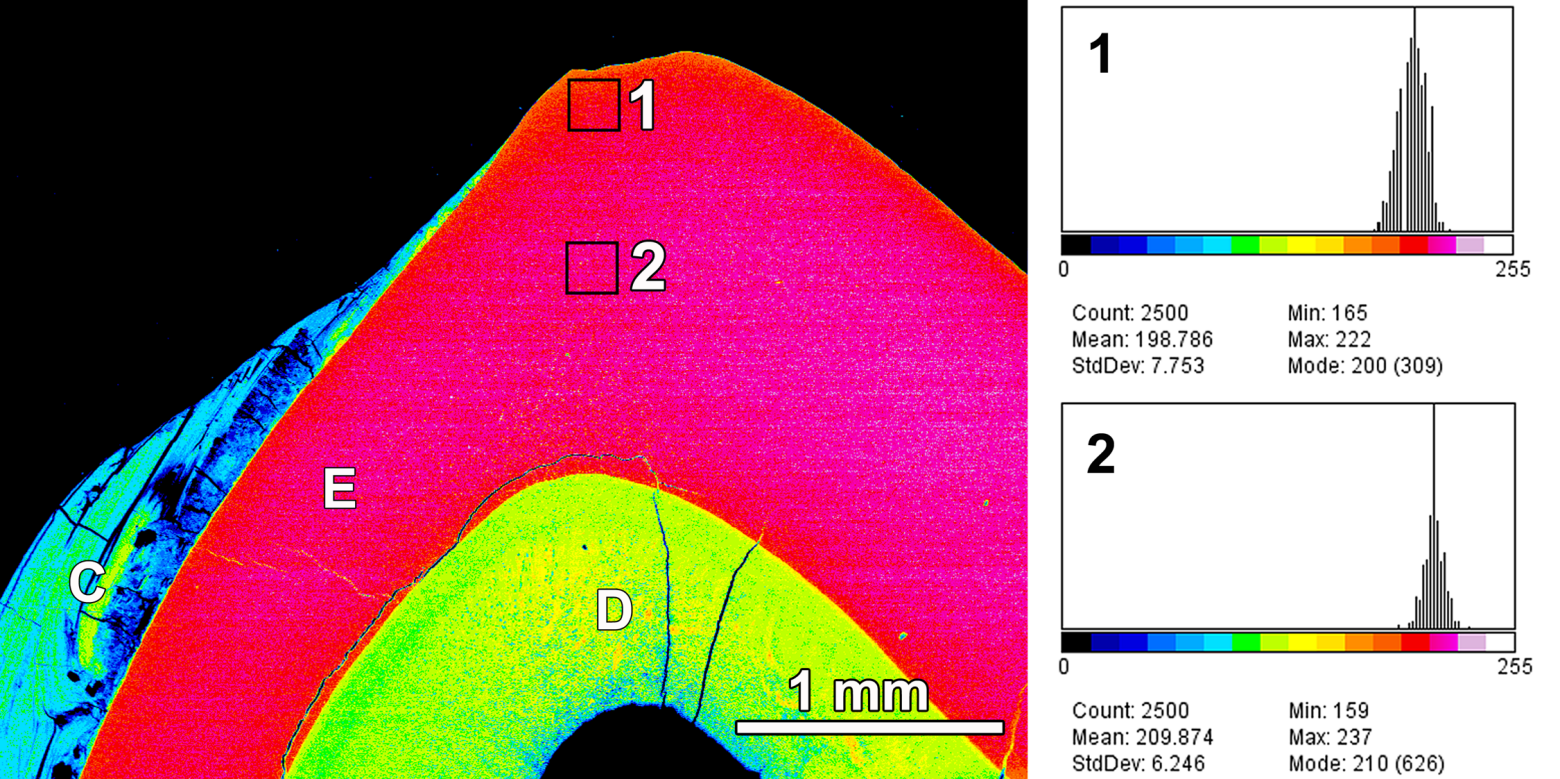
Pseudo-colour BSE image of polished cut surface of the axiobuccolingually sectioned (anterior tooth half) mandibular left third molar of individual No.2 (female *Macropus giganteus*) from a low-fluoride area, showing the degree of mineralization of enamel (E), dentine (D) and dental calculus (C). Each colour represents a grey-level band of 16 grey levels covering the grey level range from black (grey level 0) to peak white (255) as indicated by the colour bars below the histograms. The histograms show the grey level distribution, along with information on minimum, maximum, mean, and modal values and standard deviations, for the two squares indicated in the image (2500 pixels each), demonstrating that the outer enamel (square 1) is less mineralized than the deeper enamel (square 2); buccal to right of image.

Under the light microscope, *M*. *giganteus* enamel showed the typical subdivision into an inner, more extended portion featuring enamel tubules and an outer atubular portion (Fig 6A–6C). BSE imaging in the SEM revealed that in animals from the low-fluoride areas, the outer enamel was somewhat less mineralized than the deeper enamel layers (Fig 7). This hypomineralized enamel portion corresponded to the opaque outer enamel zone seen on macroscopic inspection.

**Fig 8:**
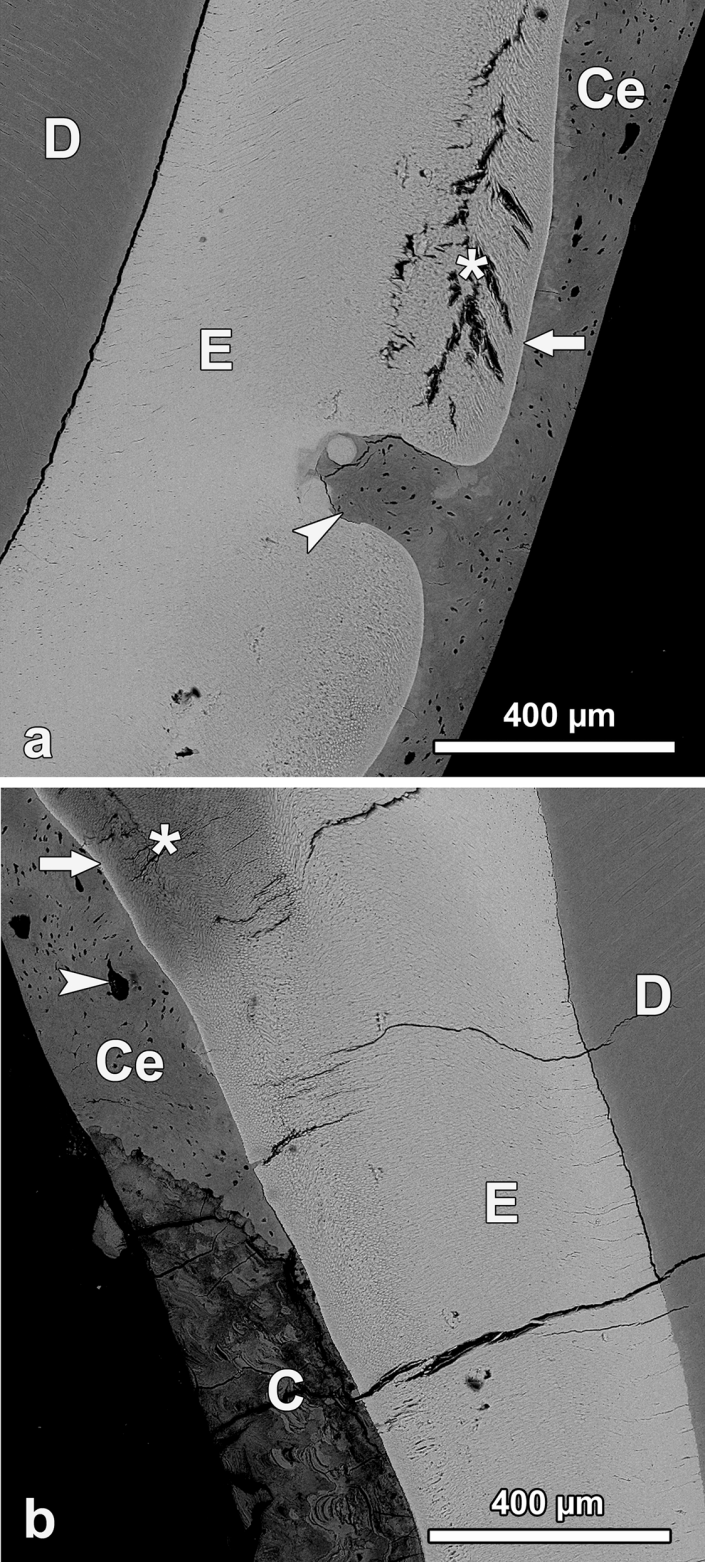
BSE images of the polished cut surface of the axiobuccolingually sectioned fluorotic mandibular left third molar (anterior tooth half) of individual No.1007 (female *Macropus giganteus*) from the high-fluoride area). (a) Buccal enamel (E) showing marked subsurface hypomineralization with cleft formation (asterisk) underneath a thin, more highly mineralized surface layer (arrow); the enamel is covered by cellular cementum (Ce); D: dentine; arrowhead: hypoplastic defect completely filled with cementum; occlusal to top of image. (b) lingual enamel (E) that is covered partly by cellular cementum (Ce) and partly by dental calculus (C); the jagged border of the cementum with the dental calculus suggests that cementum was lost from the more cervical crown portion prior to calculus deposition; D: dentine; asterisk: particularly hypomineralized outer enamel; arrow: thin surface rim of more highly mineralized enamel; arrowhead: giant lacuna in coronal cementum; occlusal to top of image.

**Fig 9:**
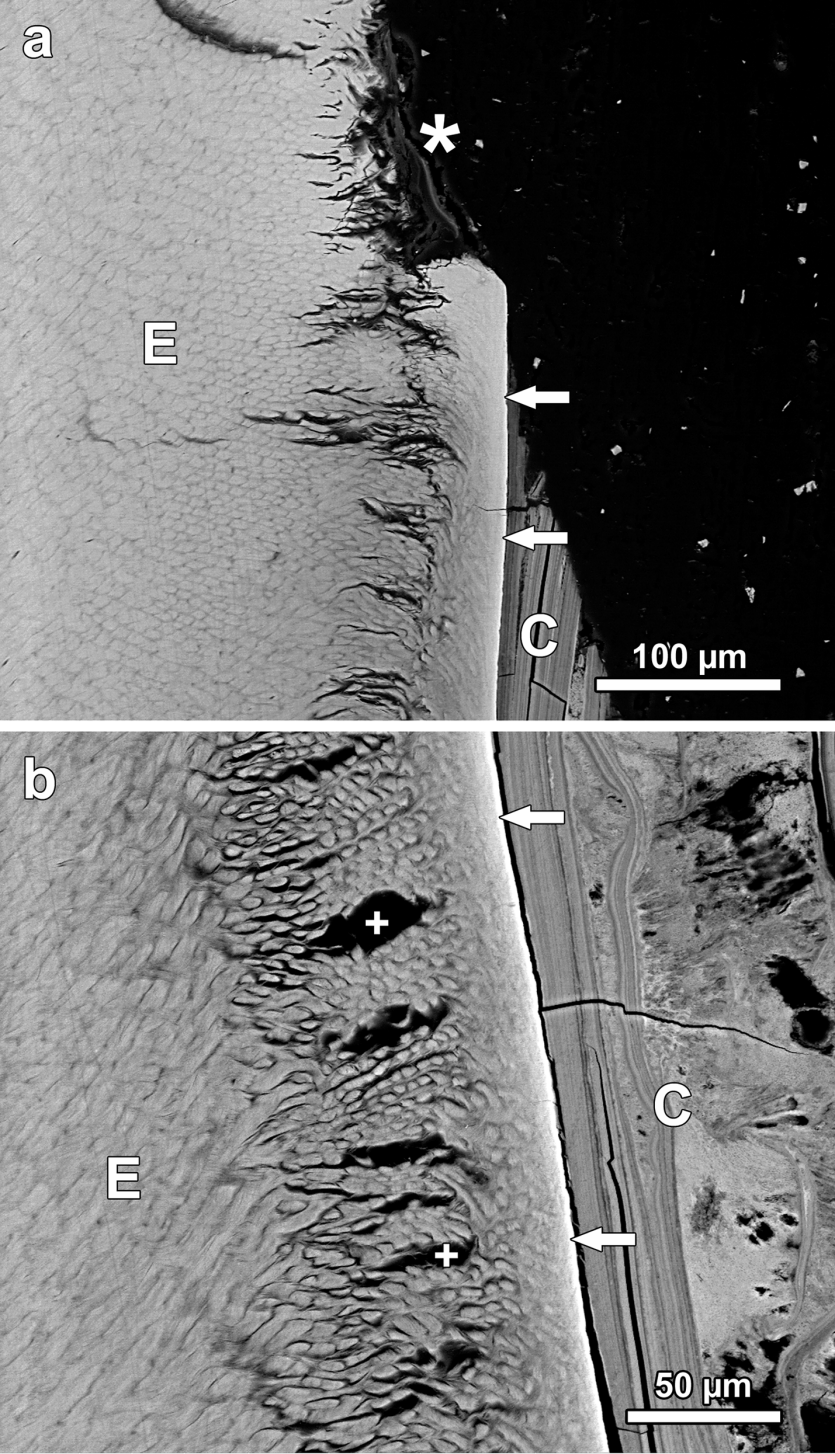
BSE images of the polished cut surface of the axiobuccolingually sectioned (anterior tooth half) fluorotic left mandibular second molar of individual No.1153 (female *Macropus giganteus*(,) from the high-fluoride area; occlusal to top of images. (a) Buccal enamel (E) exhibiting a post-eruptive defect (asterisk) due to flaking away of outer enamel. Note hypomineralized subsurface layer with numerous clefts, and thin surface rim of higher mineral content (arrows); C: dental calculus. (b) Higher magnification of buccal enamel (E) and covering layers of dental calculus (C). Note hypomineralized subsurface enamel that exhibits numerous clefts (crosses), and thin surface layer of higher mineral content (arrows); individual enamel prisms surrounded by less mineralized (darker) prism sheaths are clearly discernible. The inner (early formed) dental calculus shows a layered appearance, whereas the outer (later formed) calculus deposits exhibit an amorphous structure.

**Fig 10:**
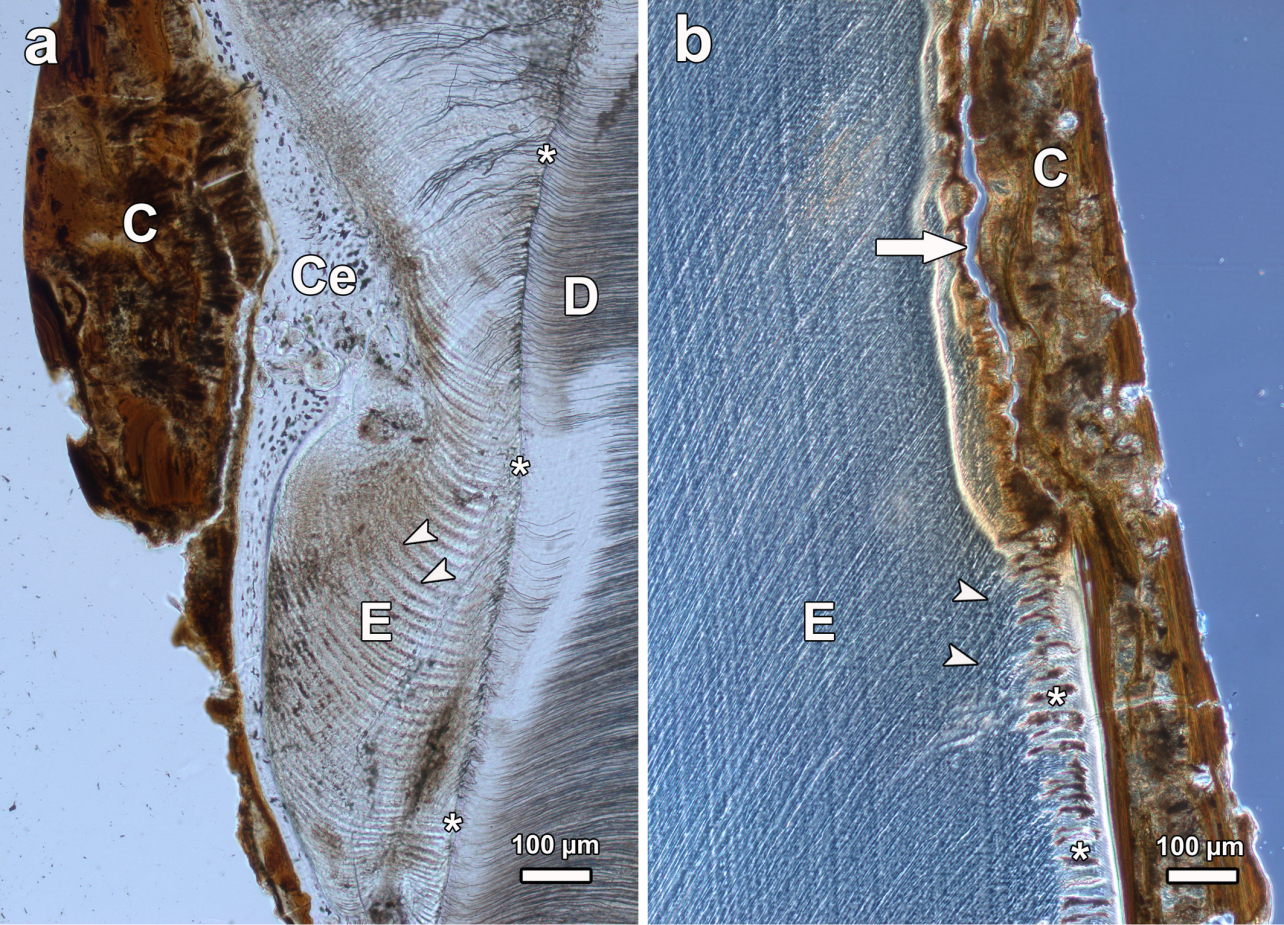
Light microscopic images of axiobuccolingual ground sections (anterior tooth half) of fluorotic molars of *Macropus giganteus* from the high-fluoride area, viewed under normal transmitted light (a) and under transmitted light with phase contrast (b). (a) Hypoplastic defect in buccal enamel (E) of the left M_3_ of individual No.1101 (female); the defect is filled with cellular cementum (Ce) that itself is overlain by dental calculus (C); note bending of incremental lines (arrowheads) in the enamel according to the margins of the defect; D: dentine; asterisks: enamel-dentine junction; occlusal to top of image. (b) Post-eruptive surface defect (arrow) in buccal enamel (E) of the left M_2_ of individual No.1153 (female). The enamel is covered by dental calculus (C). Note clefts in subsurface enamel (asterisks) in areas where the enamel surface is still intact. The incremental lines in the enamel (arrowheads) are not bent according to the margins of the defect; occlusal to top of image.

The enamel of normal and fluorotic teeth was partly covered by cementum. Corroborating previous observations [84], this coronal cementum was found to be of a cellular nature (Figs 6A, 8A and 8B). The cementocyte lacunae, whose density within the tissue varied, were mostly of a roundish to oval shape with a size of about 5 to 15 μm along the long axis. Occasionally some larger lacunae (‘giant lacunae’ [89]) were also present in the tissue (Fig 8B). While at the flanks of the tooth crowns, often only a relatively thin layer of cementum was present (Fig 6B), thicker cementum pads were found in depressions of the crown surface (Fig 6A). In cuspal areas, the cementum was frequently missing, most likely as a consequence of attrition (Fig 6A and 6B). The discontinuous nature of the cementum layer along the tooth flanks is attributed to post-eruptive loss. Evidence for this was the jagged border that could in places be observed between coronal cementum and dental calculus (shown for a fluorotic tooth in Fig 8B), denoting a local loss (chipping) of cementum prior to calculus deposition. In crown surface areas where dental calculus was present in addition to cementum, the former covered the latter (Fig 6B). The dental calculus was partly of a layered, partly of an amorphous structure (Figs 6B, 6C, 9A, 9B,10A and 10B). Due to its different structural characteristics, dental calculus could easily be distinguished from cementum in both the light microscope and the SEM (Figs 6B, 7B, 8B and 10A).

**Fig 11:**
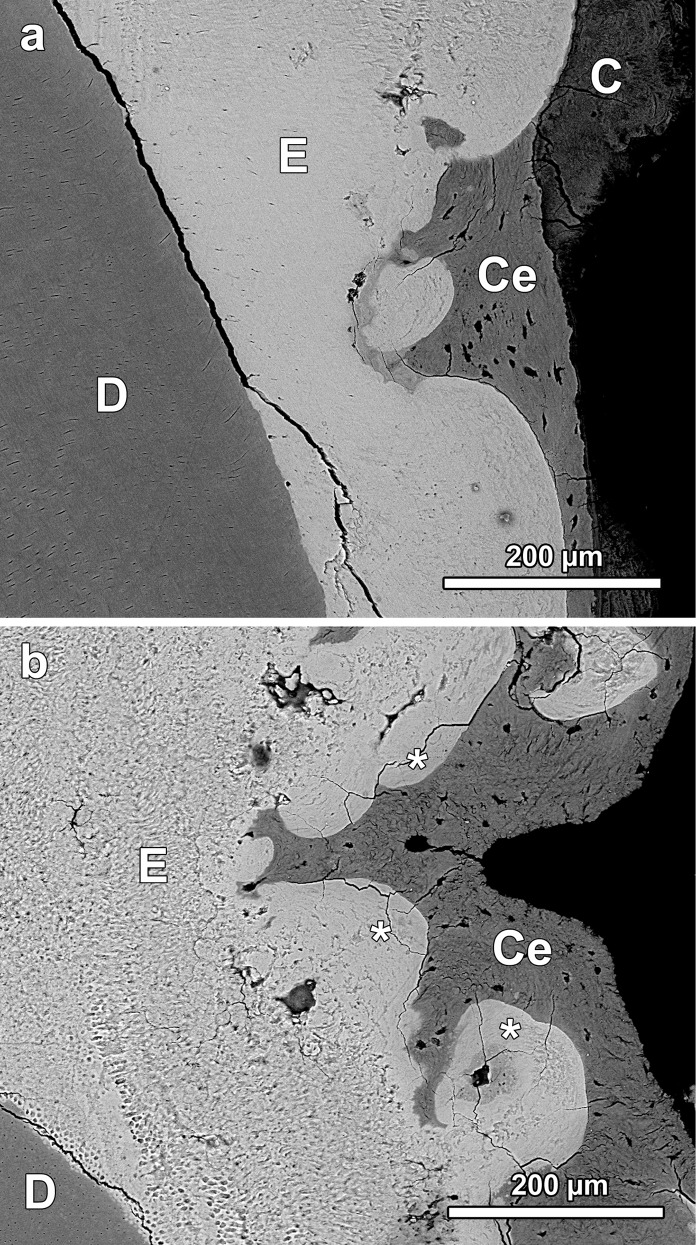
BSE images of polished cut surfaces of axiobuccolingually sectioned fluorotic mandibular third molars of *Macropus giganteus* from the high-fluoride area. (a) Hypoplastic defect in buccal enamel (E) of the left M_3_ (posterior tooth half) of individual No.1007 (female); the defect is filled with cellular cementum (Ce); C: dental calculus; D: dentine; occlusal to top of image. (b) hypoplastic defect in lingual enamel (E) of the right M_3_ (anterior tooth half) of individual No.1000 (female), the defect is also filled with cellular cementum (Ce); the outer enamel is aprismatic (asterisks); D: dentine; occlusal to top of image.

Fluorotic enamel of the eastern grey kangaroos exhibited various defects (Figs 8–11). BSE imaging in the SEM demonstrated different degrees of hypomineralization of the outer enamel. This hypomineralization extended variably wide towards the EDJ. Often, distinct patches of particularly hypomineralized outer enamel were present along a crown flank (Fig 8B). Hypomineralization of the fluorotic enamel was much more pronounced than that of the outer opaque enamel layer present in the teeth of controls from the low-fluoride areas. In regions of fluorotic tooth crowns where the original enamel surface had remained intact it became apparent that the hypomineralized outer enamel was located below a thin outermost rim of enamel with a much higher mineral content (Figs 8A, 8B, 9A and 9B). The enamel of *M*. *giganteus* from the high-fluoride area can thus be characterized as showing a distinct subsurface hypomineralization underneath a thin, well-mineralized surface layer.

Severely hypomineralized subsurface enamel regularly exhibited conspicuous clefts that could be demonstrated in the scanning electron microscope (Fig 9A and 9B) and in the light microscope (Fig 10B). Formation of theses clefts was attributed to a loss of tissue fluid originally present in the hypomineralized enamel during drying of the teeth post-mortem. The hypomineralized subsurface zones of enamel constituted zones of mechanical weakness, made evident by the fact that portions of outer enamel were frequently missing from the tooth crowns (Figs 9A and 10B). These outer enamel portions had apparently flaked off along the hypomineralized subsurface zones during mastication, thereby exposing deeper enamel layers to the oral environment. The incremental lines in the enamel did not show a bending in relation to the margins of these post-eruptive defects, which were characterized by sharp borders, steep walls, and a rough, uneven base (Figs 9A and 10B). The edges of the defect walls were sometimes rounded, which was already observable on macroscopic inspection of the post-eruptive enamel defects (Fig 5A and 5B). This was attributed to a secondary smoothing of the defect edges due to wear. Often, the post-eruptive defects had become filled with dental calculus (Fig 10B).

In addition to the post-eruptive defects, a second type of surface defect was present in the fluorotic enamel. In contrast to the post-eruptive defects, those of the second type were characterized by smooth, rounded walls (Figs 8A, 10A, 11A and 11B). The number, size and shape of these defects varied widely among the studied teeth, ranging from small isolated lesions to extensive pitting affecting large parts of the tooth crown. Under the light microscope it became apparent that the incremental lines in the enamel showed a distinct bending according to the margins of these defects (Fig 10A). This indicated that the defects were of a developmental origin, i.e., they represented cases of enamel hypoplasia. The developmental nature of these defects was further demonstrated by the fact that they had frequently become filled with cellular cementum (Figs 8A, 10A, 11A and 11B), which is deposited pre-eruptively [20].

**Fig 12:**
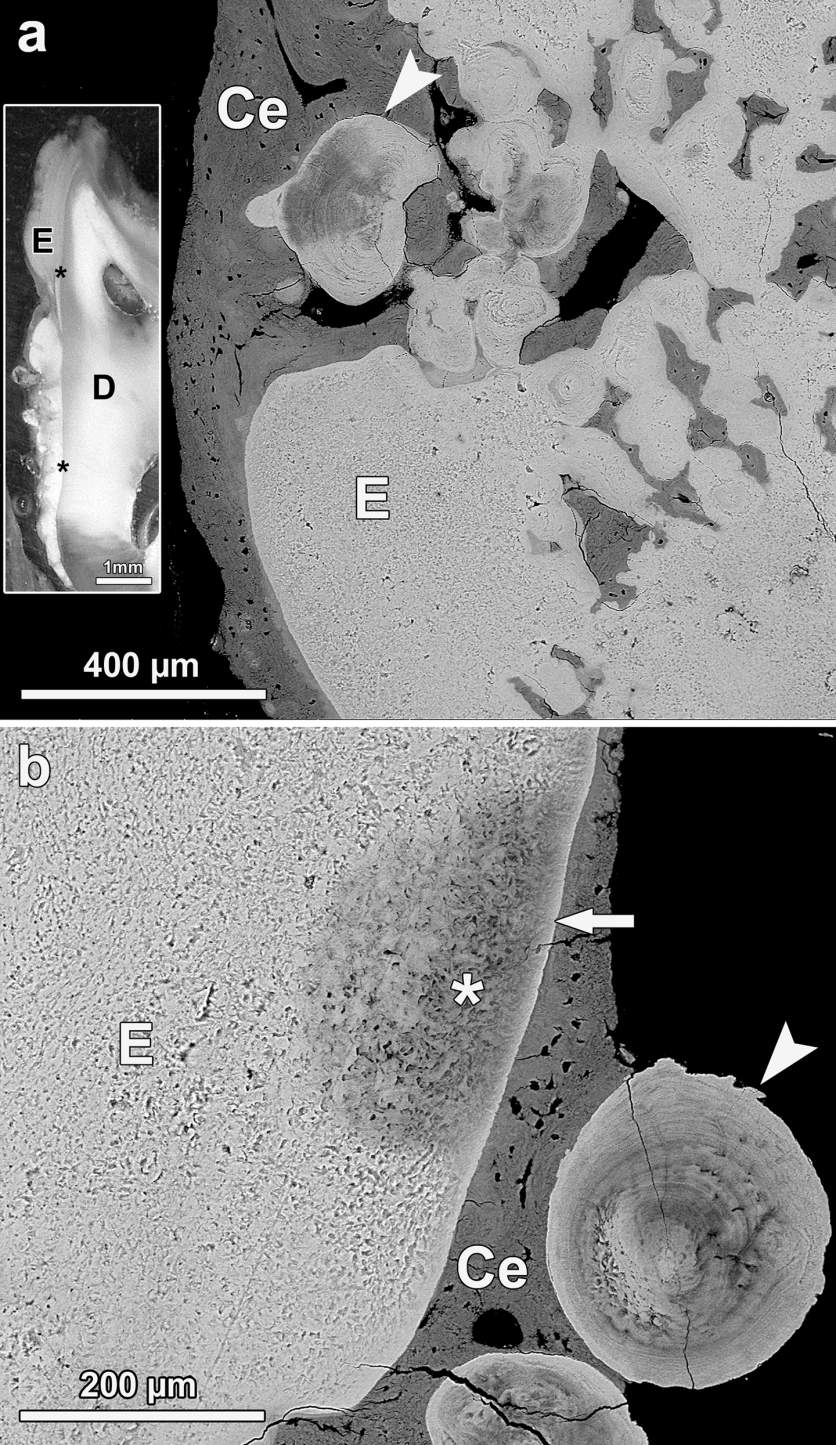
BSE images of the polished cut surface of the axiobuccolingually sectioned (anterior tooth half) fluorotic right mandibular third molar of individual No. 1000 (female *Macropus giganteus*) from the high-fluoride area. (a) Pronounced hypoplasia of buccal enamel (E). The defects are filled with cellular cementum; Arrowhead: bulbous, multilayered enamel structure exhibiting distinct hypomineralization. Occlusal to top of image. Insert: Light micrograph of buccal crown flank of the sectioned tooth. D: dentine; E: enamel; asterisks: enamel-dentine junction, Occlusal to top of image. (b) Lingual enamel showing tangentially cut multi-layered structures (arrowhead) consisting of aprismatic, hypomineralized enamel. The underlying enamel (E) exhibits a patchy subsurface hypomineralization (asterisk) underneath a thin surface layer of higher mineral content (arrow). Ce: cellular cementum. Occlusal to top of image.

The most severe case of enamel hypoplasia among the studied teeth occurred in the third molar of individual No. 1000, a young female (age at death 2.8 yr, M_4_ not yet erupted) from the smelter site with a bone fluoride concentration of 7385 μg/g dry wt. The entire crown of this tooth showed a severely disrupted enamel structure, resulting in a grossly deformed crown morphology (Figs 11B and 12A). Some hypoplastic defects extended almost down to the enamel-dentine junction, indicating a disruption of ameloblast function early during the secretory stage. The hypoplastic defects were to a large extent filled with cellular cementum (Fig 12A). Between the deep defects, bulbous structures of aprismatic enamel with a multi-layered appearance were observed (Fig 12A). Depending on the plane of section, these structures sometimes appeared as seemingly isolated spherical objects unconnected to the underlying enamel (Fig 12B). The findings in this tooth are indicative of an extensive disruption of secretory ameloblast function, causing a cessation or pronounced reduction (areas of aprismatic enamel) of enamel secretion. In addition, the enamel of this tooth showed various areas of pronounced subsurface hypomineralization, demonstrating a major disturbance also of enamel maturation (Fig 12A and 12B).

The cementum that covered the enamel in the hypoplastic areas was itself often overlaid by dental calculus (Fig 10A). Due to the occlusion of the hypoplastic enamel defects with cementum and dental calculus, their number, depth and extension along the occluso-cervical tooth axis could not be reliably assessed by external inspection of tooth crowns.

The prismatic structure of the enamel could clearly be identified in the BSE images, most prominently in the hypomineralized areas (Fig 9B). However, the enamel lining the hypoplastic defects of the fluorotic teeth typically lacked a prismatic structure (Fig 11B). Occurrence of this aprismatic (prismless) enamel indicates a loss of the distal (prism-forming) process of the Tomes’ process by the secretory ameloblasts forming this enamel. In general, the degree of mineralization of the aprismatic enamel corresponded to that of deeper enamel layers (Fig 11A and 11B).

## Discussion

To our knowledge, this is the first study analyzing the histopathological features of fluorotic enamel in a marsupial species. The principal developmental defects observed in the enamel of *M*. *giganteus* were hypoplasia and subsurface hypomineralization underneath a thin, well-mineralized surface layer. Overall, the findings in the kangaroo teeth closely match those previously reported for fluorotic enamel of domestic and wild terrestrial cetartiodactyls [24, 48, 50, 53, 63–71].

Enamel hypoplasia is caused by a functional disturbance of secretory ameloblasts and the resulting reduced enamel production [21, 22, 23, 26]. The shape, size and depth of the hypoplastic defects depend on the duration and intensity of the stress event acting on the ameloblasts, the number of cells affected, their stage of secretory activity, and their position along the forming tooth crown [22, 23, 25, 26].

Enamel hypoplasia has been experimentally produced in the central incisors of sheep receiving daily oral doses of 2, 4 or 6 mg fluoride (as NaF) per kg body weight for 21 days [24] or 4 mg fluoride per kg body weight for 26 days [64]. Histological analysis revealed that hypoplastic pitting was associated with shortening of the secretory ameloblasts, while a larger (plane-type) defect, present in one of the sheep, was associated with displacement or death of the cells [64]. Pit-type and plane-type hypoplastic defects were also produced in molar (M_3_) enamel of miniature pigs given a daily oral dose of 0.9 mg fluoride (as NaF) per kg body weight for one year [68]. Enamel hypoplasia had previously been observed in teeth of free-ranging wild boar [53] and deer [70] inhabiting fluoride-polluted areas. In the latter studies, the amount of fluoride uptake by the animals could, however, not be quantified, as was the case also for the grey kangaroos from the high-fluoride area.

More recently, hypoplastic pitting of enamel was also experimentally induced in hamster molars by injection of 40 mg NaF/kg body wt in pups [95]. Histological analysis of tooth germs suggested that in this case the hypoplastic defects were associated with a local detachment of the ameloblasts from the surface of the forming enamel and the formation of so-called sub-ameloblastic cysts.

The most profound enamel hypoplasia among the studied kangaroo teeth was observed in the M_3_ of a female kangaroo (No. 1000, estimated age 2.8 yr) that exhibited the second-highest bone fluoride concentration (7385 μg/g dry wt) of all studied animals. Similar cases of severe enamel hypoplasia affecting large parts of the tooth crown were previously observed in fluorotic teeth of wild boar [53], domestic pigs [68] and sheep [63, 64].

From the cited experimental studies [24, 64, 68, 95] it appears that enamel hypoplasia can be induced in mammalian enamel under varying fluoride exposure conditions. Type and severity of the hypoplastic defects primarily depend on the plasma fluoride levels affecting the ameloblasts and their degree of vulnerability, which differs between different (early-, mid-, late-secretory) stages of their secretory lifespan [28]. In this context it must also be considered that compared with humans (and probably other large mammals) an approximately 10-fold fluoride dose is required in small rodents to achieve similar plasma fluoride levels [28]. Possible reasons for this difference include a more rapid renal clearance of fluoride and faster bone growth rates in the latter [28].

Scanning electron microscopic and microradiographic studies suggested that the surface defects in fluorotic human enamel are of post-eruptive origin and do not constitute developmental lesions, i.e., hypoplasia [59]. This led to the concept that (human) dental fluorosis is basically the result of a fluoride impact on the maturation stage rather than the secretory stage of amelogenesis [34]. While this view may be correct, depending on the specific range of exposure conditions to fluoride and related plasma fluoride levels in humans, the present study, our previous investigations [48, 50, 53, 68], and the studies of other authors [24, 64] demonstrated that in other mammal species the secretory stage of amelogenesis is also affected by fluoride under environmentally relevant exposure conditions.

Presence of aprismatic (prismless) enamel in association with hypoplastic defects, observed in some of the fluorotic kangaroo teeth, has also been reported in fluorotic teeth of wild boar, domestic pigs and deer [53, 68, 70]. Formation of this aprismatic enamel is indicative of a markedly reduced secretory activity of the ameloblasts and a reduction of the distal portion of their Tomes’ processes [53, 68, 70]. A fully active secretory ameloblast possesses separate formation sites for interprismatic enamel (along the proximal portion of its Tomes’ process) and the prism (along its distal portion) [1, 5]. Reduction of the distal portion of the Tomes’ process in cells with reduced secretory activity will result in the presence of only a single, more or less flat, secretory surface. The crystals forming in the matrix secreted at this surface are arranged in parallel and show the orientation characteristic of interprismatic enamel. Aprismatic enamel is also formed during normal amelogenesis, *viz*. in the case of initial enamel produced prior to the establishment of the distal Tomes process by the ameloblasts and in the final enamel layer laid down in places after (normal) reduction of the distal Tomes’ process near the end of enamel secretion [5]. The hemispherical structures observed in the M_3_ of individual No. 1000 indicate that locally, groups of ameloblasts were able to continue secretion at a reduced rate, causing formation of multilayered aprismatic enamel, while neighbouring ameloblasts had already prematurely ceased matrix production.

BSE-imaging of molars from the control individuals revealed that in *M*. *giganteus* the outer (opaque) enamel is less mineralized than the deeper enamel layers that appear translucent on macroscopic inspection. This corroborates previous findings in the eastern grey kangaroo and other macropodid species by Palamara et al. [96]. These authors used hardness testing to demonstrate that the opaque outer enamel was softer (less mineralized) than the translucent inner enamel. Further to this, they showed that the division of the enamel into translucent and opaque zones was not identical to the tubular/atubular division. Transmission electron microscopy demonstrated that the translucent enamel consisted of well defined prisms and well-packed crystals, while the opaque enamel was characterized by inter-crystalline voids and randomly orientated, loosely packed crystals. It was concluded that the opacity of the outer enamel was caused by scattering of light from the intercrystalline voids [96]. Regarding the functional significance of the opaque outer enamel it was suggested that the presence of a softer outer enamel layer may accelerate the achievement of a fully functional, broad contact between occluding teeth by rapidly wearing away of crown tips, thereby helping to maintain the functional spatial relationship between tooth crowns under the conditions of molar progression and interstitial wear [96].

The subsurface hypomineralization underneath a thin surface rim of high mineral content seen in fluorotic enamel of *M*. *giganteus* was previously also observed in fluorotic enamel of humans and other placental mammals [32, 48, 53, 55, 66, 68, 71, 97]. It has been demonstrated that the well-mineralized surface rim forms before tooth eruption [29, 98, 99]. Regarding differential diagnosis, it has been concluded that none of the histological features of fluorotic enamel, including the subsurface hypomineralization discussed here, can be considered pathognomonic of dental fluorosis [33]. With respect to the clinical diagnosis of human dental fluorosis, other authors, however, stated that it is most unlikely that enamel changes of non-fluoride origin will constitute a differential diagnostic problem [32]. In the present study, the diagnosis of dental fluorosis in the eastern grey kangaroos is clearly supported by the bone fluoride data.

Based on the observation that erupted fluorotic enamel resembles normal, partially mineralized maturation stage enamel it was hypothesized that the hypomineralization of fluorotic enamel is caused by an impairment of ameloblast function during the maturation stage [31]. In line with this view, experimental studies in pigs [67] and sheep [65] later demonstrated that the typical hypomineralization of fluorotic enamel can be induced if fluoride affects the maturation stage only. However, in the sheep study, hypomineralization increased in depth and severity when fluoride was also given during the secretory stage [65]. This suggests a fluoride-effect already on the early stages of enamel mineralization occurring during the secretory stage, followed by further disturbance of the mineralization process during the maturation stage. This observation notwithstanding, the hypomineralization of fluorotic enamel is currently regarded to be basically due to an impairment of the maturation-stage of amelogenesis, during which the majority of mineral accretion takes place [28, 34, 50, 53, 68]. Several studies have shown that higher fluoride levels in plasma and tissue fluids interfere with the enzymatic degradation of the enamel matrix and subsequent enamel mineralization [28, 34]. However, the exact way in which fluoride impairs enamel maturation is still unknown and currently several potential mechanisms are discussed.

A recently proposed model [100] is based on the known release of large amounts of H^+^ ions that accompanies the massive precipitation of hydroxyapatite during the maturation stage, and the differential permeability of the cell membrane for F^−^ and HF. It is argued that the acidification of the extracellular milieu around the ameloblasts promotes the conversion of F^−^ to HF that (contrary to F^−^) can easily diffuse into the cells. At the neutral pH of the cytosol, the HF then dissociates into H^+^ and F^−^. As the cell membrane is nearly impermeable to F^−^ [101], the fluoride anion cannot leave the ameloblast, and toxic intracellular concentrations can eventually build up if fluoride is present in larger amounts in the ameloblast environment. Based on different lines of evidence, Sharma et al. [100] conclude that the resulting cellular stress response compromises ameloblast function, in particular the synthesis and secretion of proteins, including those responsible for the enzymatic degradation of enamel matrix proteins. In consequence, larger amounts of matrix protein are retained in the enamel, which in turn inhibits its full maturation and leads to the characteristic hypomineralization of fluorotic enamel.

Along a similar line of reasoning it was recently hypothesized that the increased release of H^+^-ions associated with the fluoride-induced massive hydroxyapatite precipitation in forming enamel impairs ameloblast function and ion transport processes [99]. It was further suggested that the formation of a well-mineralized surface layer under maturation-stage ameloblasts could act as a physical barrier, impeding the diffusion of proteins and mineral ions into the subsurface enamel and thus leading to its characteristic hypomineralization [99].

With respect to the diagnosis of dental fluorosis in the eastern grey kangaroo, it must be considered that the hypomineralized (opaque) outer enamel layer is a regular feature of normal teeth [99]. However, compared to the teeth of the animals from the low-fluoride areas, the degree of hypomineralization and, in consequence, the opacity of the fluorotic enamel was typically much more pronounced. The greater porosity of fluorotic compared to normal enamel of *M*. *giganteus* teeth also explains why the latter enamel shows a whitish appearance, while the former is typically discoloured due to penetration of staining substances from the oral cavity into the large intercrystalline voids. Thus, while the macroscopic appearance of the outer enamel in normal teeth might on first sight be confused with mild dental fluorosis as described for humans [32] and other placental mammals [40], a closer inspection of the teeth will normally allow a distinction between normal and fluorotic *M*. *giganteus* enamel.

It may be hypothesized that the naturally occurring hypomineralization of the outer enamel in *M*. *giganteus* and other macropodids renders their teeth particularly susceptible to fluoride. According to Palamara et al. [96] crystal size does not differ between translucent and opaque regions of macropodid enamel. Information on crystal size in fluorotic marsupial enamel is presently not available, and studies comparing crystal dimensions, orientation and packing in sound opaque and hypomineralized fluorotic enamel of marsupials are therefore encouraged.

In conclusion, the present study has demonstrated that fluorotic enamel in eastern grey kangaroos exhibits developmental and post-eruptive defects that closely match those previously described in placental mammals. The observed defects denote a fluoride-induced disturbance of ameloblast function during both the secretory stage and the maturation stage of amelogenesis. The spectrum of macroscopic and histopathological lesions observed in fluorotic enamel of *M*. *giganteus* leads us to conclude that there exist no principal differences in the pathogenic mechanisms of dental fluorosis between marsupials and placental mammals. The regular (physiological) occurrence of a hypomineralized, opaque outer enamel layer in the teeth of the eastern grey kangaroo and other macropodids must be considered in the differential diagnosis of dental fluorosis in these species.
